# Supplementary material for: Identification of photoperiod-induced specific miRNAs in the adrenal glands of Sunite sheep (Ovis aries)
Source: Front Vet Sci. 2022 Jul 22;9:888207. doi: 10.3389/fvets.2022.888207 (PMC9354845; doi:10.3389/fvets.2022.888207)
Supplement: Supplementary file 1 [file Data_Sheet_1.ZIP › Supplementary file/miRDeep2 file.zip!/miRDeep2 file/Know_Structure/LP42A3/oar-mir-30c.pdf]

miRBase precursor : oar-mir-30c
Total read count : 83033
oar-miR-30c read count : 82861
remaining reads : 172

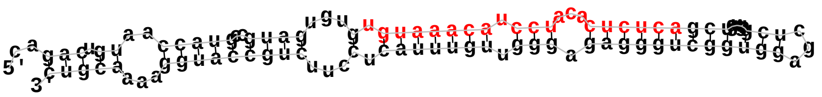

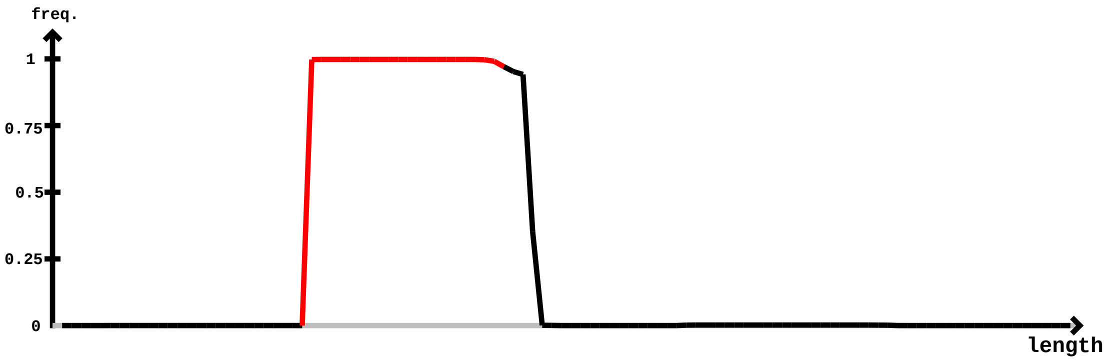


```
                                             oar-miR-30c
5'- cagacuguaaccaugccguagugugu guaaacauccuacacucucagcugugagcucgagguggcugggagaggguuguuuacuccuucugccauggaaaacguc   -3'    exp
```

oar-miR-30c

```
cagacuguaaccaugccguagugugacuguaaacauccuacacucucagcugugagcucgagguggcugggagaggguuguuuacuccuucugccauggaaaacguc
................................................................cugggagaggguuguuuacucc...................   92   0   seq
................................................................cugggagaggguuguuuacuccu..................   5    0   seq
................................................................cugggagaggguuguuuacuccuu.................   2    0   seq
.................................................................ugggagaggguuguuuacu.....................   3    0   seq
.................................................................ugggagaggguuguuuacuc....................   6    0   seq
.................................................................ugggagaggguuguuuacucc...................   19   0   seq
```
